# Promoting Heart Health: Raising Screening Awareness at a Brisbane Medical Center

**DOI:** 10.7759/cureus.62183

**Published:** 2024-06-11

**Authors:** Reet Dhaliwal (Mangla), Steven Altawil

**Affiliations:** 1 Family and Community Medicine, Oceania University of Medicine, Brisbane, AUS; 2 General Practice, Altawil Medical Centre, Goodna, AUS

**Keywords:** interventions, compliance, knowledge, screening, cardiovascular disease

## Abstract

Background

Atherosclerotic cardiovascular disease (CVD) is a largely preventable, chronic, and progressive medical condition. There appears to be a general lack of knowledge about CVD prevention in the community. This pilot study was carried out to investigate the level of knowledge of CVD prevention among patients visiting a general practitioner (GP) practice in Brisbane.

Aim

To investigate the level of knowledge of CVD prevention among people visiting a local medical clinic in Brisbane, and to identify the factors responsible for any knowledge deficits.

Material and methods

A cross-sectional survey was conducted among Brisbane residents aged 45 years and older visiting a local medical center. This study surveyed 105 Australian individuals via an online survey or a hard copy questionnaire for those without online access. A combination of closed-ended questions and multiple-choice questionnaires was utilized to collect the data.

Questions were formulated to assess the level of knowledge of CVD prevention among this demographic, exploring the subjects' awareness of the screening program, their adherence, factors behind non-adherence, and understanding the influence of education level and occupation on adherence.

Results

A total of 105 Brisbane adults completed the survey, of which 56 (53.3%) were male. The study found that 61 (58.1%) of the participants were aware of the CVD prevention screening program, but only 22 (21.9%) were compliant with it. Twelve (11.4%) participants attended these preventive screenings less frequently than recommended, while the remaining 69 (66.6%) had never undergone cardiovascular health checks.

Conclusion

Analysis of data from this population suggests that there is a knowledge deficit among Brisbane residents regarding CVD prevention programs. Enhanced efforts by clinicians to make patients aware of these screening tools and to employ early intervention strategies, especially lifestyle choices at an earlier age, would help lower the burden of CVD.

## Introduction

Atherosclerotic cardiovascular disease (CVD) remains the leading cause of mortality, morbidity, and overall healthcare expenditure globally, despite significant improvements in outcomes in recent years. According to the 2016 WHO report, ischemic heart disease was responsible for approximately 9 million deaths worldwide [1,2].

One Australian has a heart attack or stroke every four minutes. Many people may not be aware of their risk factors for heart disease, and some risk factors, like high blood pressure or high cholesterol, can be silent [3]. Approximately 1.4 million Australians have an increased chance of having a heart attack or stroke in the next five years [4,5], yet many are unaware of this risk. One-fifth of Australians aged 45-74 years have a high chance of having a heart attack or stroke in the next five years [6].

Promoting cardiovascular health awareness among Australian adults can empower them to make informed decisions about their health and improve their quality of life. Early detection is critical as it helps prevent or delay the onset of complications associated with heart diseases [3-6].

The Australian Heart Foundation (AHF) recommends age-specific guidelines for heart health screenings. Anyone aged 45 and over, or 30 and over for Aboriginal and Torres Strait Islander peoples, should have a regular heart health check annually with their doctor. This check should include assessments of weight, BMI, blood pressure, and cholesterol levels, along with the collection of patient information to identify CVD risk factors such as diabetes status, alcohol intake, smoking status, medication history, and family history to assess their risk of having a cardiovascular event in the next five years [4-6]. Interventions can be implemented depending on the risk estimation: lifestyle factor modifications for low risk, lifestyle factor modifications and medication management for moderate risk, and more intense interventions including specialist input for high-risk individuals [7].

The purpose of this pilot study is to identify the level of knowledge among Brisbane residents about their cardiovascular health awareness and the use of recommended disease prevention screening programs. The underlying proposition for the study is that adults in Australia exhibit insufficient knowledge about cardiovascular health risk screening programs. It is also hypothesized that higher levels of education and related occupations directly correlate with CVD screening awareness.

## Materials and methods

This research study utilized a survey-based approach to explore the level of knowledge among Brisbane's adult population aged 45 and over about their awareness of cardiovascular risk screening programs. The study was carried out within a Brisbane suburb and involved 105 randomly chosen participants. Data was collected over a period of approximately three months starting from February 12, 2024. Data collection involved distributing questionnaires to patients and their relatives attending the Altawil Medical Centre in Goodna, Brisbane.

The survey included a total of 10 questions that delved into the participants' level of awareness regarding the CVD screening program, their compliance with the program, and the factors contributing to non-compliance. The survey also sought to establish any potential correlations between participants' education levels, occupations, and their adherence to the CVD screening program. It featured a mix of closed-ended questions, which required specific responses, and multiple-choice questions, offering a selection of predefined options. These questions were distributed through an online survey platform, and printed copies of the survey were made available to accommodate individuals without internet access. A sample of the questionnaire is provided in the appendices (Appendices 1-2).

Individuals 45 years and over who were able to understand English and attended the GP clinic in Brisbane were chosen to participate in this study. Age group brackets were distributed as follows: 45-59, 59-69, and 70 & above. The level of education attained by participants was also included in the questionnaire, encompassing primary school, high school, trade/vocational courses, and university degrees - these educational tiers are standard in Australia [7]. In the survey, participants were presented with six options for occupation: medical, teaching, engineering, trade/transport, others, and unemployed/unable to work. These categories were carefully chosen to cover a wide range of major industries. For instance, the medical category encompassed professions such as doctors, nurses, dentists, optometrists, pharmacists, and even receptionists working within medical establishments. Similarly, the engineering category included professionals such as engineers, mine workers, draftsmen, technicians, and machinists. The "unemployed/unable to work" category referred to individuals who were not currently employed in any capacity. Participants were asked whether they were aware of the cardiovascular screening program and their compliance status, with options including following the guidelines annually as per the Australian Heart Association, following the guidelines less frequently, or not undergoing the recommended health screening. An analysis was conducted to identify the factors contributing to non-compliance with the recommended guidelines for CVD screening. In the survey, a section was included that gathered information about the primary sources of awareness for participants. The options provided are based on common sources available for raising awareness in communities, including the participants' own education and occupation, recommendations by their general practitioners, input from family and friends, as well as community-based sources such as banners, seminars, and information sheets. Gathering this information will help us understand the various channels through which participants receive information and make decisions.

Informed consent was obtained from the participants, and anonymity was guaranteed. Internal Review Board approval for this project was granted by the Oceania University of Medicine (IRB HREC24_014). Once data collection was completed, all analyses were conducted using Excel. Information was collected and stored securely by the principal investigator. The initial step of our analysis involved a comprehensive examination of the awareness levels regarding the program across various age groups. This investigation was conducted to accurately determine the compliance status among Brisbane residents and to compare it against the national compliance rate.

Additionally, this research study delved into the influence of education on compliance rates, aiming to understand how varying levels of education impact individuals' adherence to guidelines or regulations. The study also explored the correlation between participants' occupations and their compliance rates, recognizing the significant connection between education and occupation. Lastly, the analysis aimed to capture comprehensive insights into the sources through which individuals become aware of health awareness programs, shedding light on the most effective channels for disseminating such information.

## Results

Of the 105 participants surveyed, 56 (53.3%) were male, and 48 (45.7%) were female. In terms of awareness, 61 participants, constituting 58.1% of the sample, were aware of the cardiovascular screening program, whereas 41.9% were not. The highest number of participants were from the age group 45-59 (68, 64.8%), followed by 27 (25.7%) from the age group 59-69, and the remaining 10 (9.5%) from the age group 70 and above (Table 1).

**Table 1:** Demographics of the participants. CVD: Cardiovascular disease.

Options		
Awareness of the participants about the CVD health screening program	Numbers	Percentage (%)
Yes	61	58.1
No	44	41.9
Age of participants		
45-59	68	64.8
59-69	27	25.7
70 and above	10	9.5
Highest education completed by participants		
Primary school	3	2.9
High school	45	42.9
Certificate or trade/vocational course	18	17.1
Bachelor’s degree	29	27.6
Master’s degree or higher	10	9.5
Occupation of the participants		
Medical	9	8.6
Teaching	3	2.9
Trade/Transport	15	14.3
Engineering	4	3.8
Unemployed/Unable to work	19	18.1
Others	55	52.4

Table 1 also demonstrates the education level of participants, revealing the majority of surveyed participants completed high school (42.9%), followed by those with bachelor's degrees (29, 27.6%). Only 10 (9.5%) participants completed a master’s degree or higher, while 21 (20.0%) completed only primary school or trade courses.

In terms of occupations, 19 participants were unemployed, accounting for 18.1% of the total encounters, followed by trades/transport employees (15, 14.3%). There were nine individuals from medical backgrounds, constituting 8.6% of the surveyed population, while those in engineering made up 3.8% of the total surveyed cohort, followed by teaching (2.9%). The majority of participants, 55 (52.4%), were from other occupational groups and chose not to specify their occupation.

Interestingly, only 23 of the total surveyed individuals (21.9%) were compliant with the annual screening guidelines, even though 61 (58%) were aware of the tool. Of the total group, 11.4% participated less frequently, and 66.6% had never undergone cardiovascular health checks (Table 2).

**Table 2:** Frequency of participants undergoing this recommended CVD screening. CVD: Cardiovascular disease.

Options	Numbers	Percentage (%)
Yes, annually	23	21.9
Yes, but less frequently	12	11.4
No	70	66.6

Table 3 demonstrates the major factors behind the low compliance rate for this screening program. It was observed that a significant number of study participants, 17 (16.1%), were not undergoing these preventative checks due to their busy life schedules, while health insurance/financial issues contributed 5.7% to this low rate. Various other reasons, such as a lack of understanding of the disease process, difficulty in accessing healthcare, and various cultural and background factors, contributed approximately 21% to this non-compliance rate.

**Table 3:** Factors behind non-compliance.

Options	Numbers	Percentage (%)
Not aware	59	56.1
Busy life schedule	17	16.1
Cultural and other background factors	1	0.9
Health insurance or other financial issues	6	5.7
Other reasons	22	20.9

Participants aware of the CVD screening program selected various sources for their knowledge regarding this program. General practitioners (GPs) were the most common source of information and were chosen by 25 (23.8%) of the surveyed population, followed by family or friends (15.2%), self-education (9.5%), community resources (3.8%), and occupational background (2.9%) (Table 4).

**Table 4:** Primary source of awareness.

Options	Numbers	Percentage (%)
Not aware	47	44.8
GP recommended it	25	23.8
Family and friends	16	15.2
Own education	10	9.5
Community resources	4	3.8
Own occupations	3	2.9

## Discussion

Our study delved into demographic factors, reasons for non-compliance, and sources of awareness among participants who were aware of the cardiovascular screening program at a Brisbane GP clinic. Given the importance of heart screening programs in early detection and prevention, it is helpful to identify the current proportion of people following these guidelines and the factors responsible for non-compliance with screening. Understanding non-compliance will help us develop more effective strategies to increase the compliance rate of cardiovascular disease screening, which will further help reduce the morbidity, mortality, and healthcare costs associated with this disease [1-6]. According to AHF and WHO guidelines, baseline screening, including BMI, blood pressure, and cholesterol levels, should start as early as age 20 [5,7]. Thus, annual screening, including BMI, BP, lipid profile, family history, lifestyle factors, and medication assessment, should commence by the age of 45 at the latest. High-risk individuals, such as Aboriginal and Torres Strait Islanders, should be considered for annual screening at earlier ages [5-8].

Our study revealed several common reasons for non-compliance, including lack of trust in the screening program, cultural or background influences affecting perceptions of healthcare, financial barriers such as lack of health insurance, and the impact of busy lifestyles on scheduling and attending health checks. Additionally, other factors such as fear of receiving negative results, lack of awareness about the importance of screening, and discomfort with medical procedures were also observed. These were included in the 'other factors' category. In terms of previous studies detecting risk factors for CVD, Mageswaran N et al. conducted a cross-sectional study in Malaysia among individuals under 50. They found that hypertension and obesity are significant risk factors for CVD, yet no interventions were in place for these individuals due to a lack of health literacy about their association with CVD risk [7-15]. Hagan NA et al., during their cross-sectional studies, found that social determinants, for example, low-income status, education level, and occupation, significantly impact people’s knowledge and compliance towards CVD screening programs [7, 9-13]. Another study conducted in Korea by Lee H et al. concluded that CVD health screening was associated with lower rates of mortality and lower healthcare utilization and costs related to this disease [13,14].

Our results also found a similar impact of social determinants on the awareness and compliance rates for CVD prevention measures, noting that the effectiveness of early interventions has been supported by various studies in the literature [7,11,15,16]. Among the 105 participants analyzed during this study, the non-compliance rate of the unemployed was 42%, followed by those in trades and transportation at 28%. Medical professionals, teachers, administrators, lawyers, economists, and other professionals had high compliance rates. The prevalence of the CVD prevention program is widely known among those with higher levels of education. Of those compliant with or aware of the screening program, 98% had completed at least high school education. In comparison, the non-compliance rate was very high among those who had only completed primary school and trade courses.

Our study should encourage clinicians to make people aware of CVD preventative programs during their visits and employ brief opportunistic interventions to encourage people to improve their health literacy. Early interventions, especially at a younger age, and particularly advocating for lifestyle change, will help lower the burden of CVD [15-17]. Heeding the results of this study will benefit the community, given the significant number of Australian adults at high risk and the overall burden of CVD in Australia, as well as the proven worth of preventative programs.

An extended goal is to alleviate the burden of CVD in Australia and reduce healthcare costs. In 2019-20, an estimated 9.1% of the total allocated expenditure in the Australian health system ($12.7 billion) was attributed to CVD, second only to musculoskeletal disorders at $14.6 billion [1,18]. However, CVD remains the leading cause of death in Australia.

Although numerous screening guidelines have been developed for early detection and prevention, the Australian Bureau of Statistics and the AHF showed that the compliance rate for these screening programs is less than 50%. However, the compliance rate in our study was even lower, at 21.9% [4,13,19].

Verma KP et al. have strongly emphasized that all Australians must know and understand their absolute cardiovascular risk, which is enhanced by the use of CVD prevention tools [20-22].

As for all studies, there are limitations in this research study. Firstly, this study is limited to a medical center in a Brisbane suburb and the sample size is small. Thus, there is limited variation in demographic, socioeconomic statuses, or cultural backgrounds. We particularly note the significant variation across regions and ethnic groups in Australia, with Aboriginal and Torres Strait Islanders being more than three times as likely as non-Indigenous people to have a CVD risk, according to the Australian Bureau of Statistics [21,22,23]. It is imperative to acknowledge and address these limitations appropriately to ensure the validity and reliability of the study’s results [24,25].

## Conclusions

This pilot study suggests that there is a deficiency in knowledge of CVD risks and preventive measures among adult Australians, which should be addressed to help reduce the burden of this disease. Furthermore, the study found that many lifestyle factors, such as busy life schedules, cultural and background factors, and financial factors, significantly impacted the rate of compliance with this CVD screening program. This insight will help develop targeted education strategies. Understanding that the level of education and type of occupation directly relate to people’s knowledge of prevention strategies is crucial. Knowledge gained from general practitioners, family, friends, and community sources also plays a significant role. Enhanced efforts by clinicians to make people aware of these screening tools and to employ early interventions, especially at younger ages by advocating for healthy lifestyle choices, would help lower the burden of this disease. Additionally, our study highlights the important role of the general practitioner in educating the public about the CVD prevention tool.

## Figures and Tables

**Figure 1:**
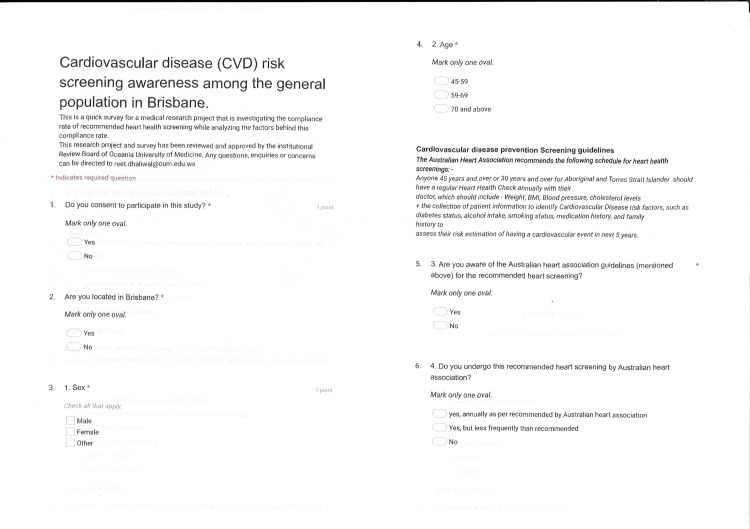
Questionnaire part 1.

**Figure 2:**
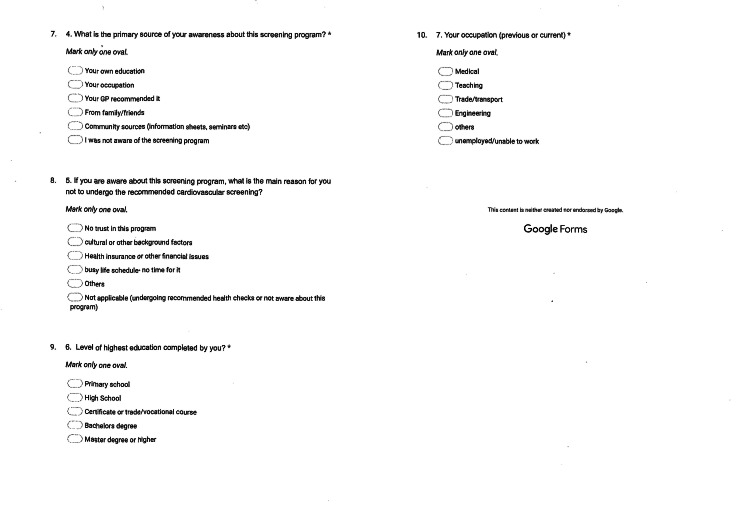
Questionnaire part 2.

## Appendices

Appendix 1 

Appendix 2 
